# Melittin Inhibits Hypoxia-Induced Vasculogenic Mimicry Formation and Epithelial-Mesenchymal Transition through Suppression of HIF-1α/Akt Pathway in Liver Cancer

**DOI:** 10.1155/2019/9602935

**Published:** 2019-04-01

**Authors:** Qunwei Chen, Wanfu Lin, Zifei Yin, Yong Zou, Shufang Liang, Shanming Ruan, Peifeng Chen, Shu Li, Qijin Shu, Binbin Cheng, Changquan Ling

**Affiliations:** ^1^Department of Oncology, Zhejiang Provincial Hospital of Traditional Chinese Medicine, Zhejiang 310006, China; ^2^Department of Traditional Chinese Medicine, Changhai Hospital, Second Military Medical University, Shanghai 200433, China; ^3^Department of Gastroenterology, Baoshan Branch, Shuguang Hospital Affiliated to Shanghai University of Traditional Chinese Medicine, Shanghai 201900, China

## Abstract

In this study, we investigated whether melittin could suppress hypoxia-induced vasculogenic mimicry (VM) formation in liver cancer and explored the underlying mechanisms. Melittin significantly inhibited the proliferation of liver cancer cells with or without CoCl_2_ presence. Melittin also significantly inhibited CoCl_2_-induced migration, invasion, and VM formation of liver cancer cells. CoCl_2_ treatment suppressed the expression of E-cadherin and elevated the expression of N-cadherin and Vimentin. Melittin reversed the changes in the protein and mRNA levels of these epithelial-mesenchymal transition (EMT) markers. CoCl_2_-induced accumulation of HIF-1*α* increased the level of phosphorylated Akt and upregulated the expression of VEGF and MMP-2/9. Melittin decreased the HIF-1*α* level and thereby suppressed the levels of p-Akt, VEGF, and MMP-2/9. In addition, the inhibitor of PI3K/Akt also suppressed CoCl_2_-induced EMT and liver cancer cells migration, and the activator of Akt, SC-79, partly blocked the effect of melittin on CoCl_2_-induced EMT and liver cancer cells migration. In the xenograft tumor model in nude mice, melittin treatment significantly suppressed the tumor growth, VM formation, and HIF-1*α* expression in the tumor. In conclusion, this study indicates melittin may inhibit hypoxia-induced VM formation and EMT in liver cancer through inhibiting HIF-1*α*/Akt pathway.

## 1. Introduction

Liver cancer is one of the most common malignant tumors worldwide, especially in China. Metastasis is still the main cause for the treat failure and poor prognosis of liver cancer patients, even those with resectable small tumors [1]. As a typical tumor with rich blood perfusion, angiogenesis plays a crucial role in the growth, migration, and invasion of liver cancer cells. However, the agents targeting the angiogenesis in liver cancer have not reached original expectancy.

Vasculogenic mimicry (VM), which is known as the formation of tumor cell-lined microvascular channels independent of endothelial cells, is considered to lead to the failure of vascular-targeted therapy and tumor metastasis [2]. In liver cancer, patients with VM show higher metastasis rate, shorter overall survival time, and worse prognosis than those without VM, and VM correlates with higher recurrence rate after liver transplantation [3, 4]. Moreover, in vitro study showed that liver cancer cells with high metastatic potential are more likely to form VM than those with low metastatic potential [5].

Formation of VM interprets the reason for the poor effect of antiangiogenesis therapy in liver cancer. On the other hand, overgrowth of tumor or angiogenesis inhibitors produces a hypoxia microenvironment, which is an important feature of rapidly proliferating malignant tumors, induces the upregulation of hypoxia-inducible factor 1 (HIF-1) and its target genes, including vascular endothelial growth factor (VEGF), matrix metalloproteinase (MMP)-2 and MMP-9, and thereby promotes VM formation and increased tumor invasion and metastasis ability [6–11]. Furthermore, HIF-1 also regulates the expression of a variety of proteins, which play vital roles in many aspects of cancer biology.

Melittin, the main component of bee venom, has extensive biological activities and pharmacological effects [12]. Our previous studies have confirmed that melittin could inhibit proliferation, migration, and invasion and induce apoptosis of liver cancer cells through Rac1-dependent pathway [13]. Further studies showed that melittin is also able to inhibit the proliferation and migration of vascular endothelial cells and downregulate the expression of the proangiogenic factor -- VEGF and basic fibroblast growth factor (bFGF) [14]. These results suggest that melittin plays an important role in the inhibition of angiogenesis and liver cancer metastasis. However, whether melittin may also suppress the VM formation in liver cancer has not been elucidated. Therefore, we used Cobalt chloride (CoCl_2_), which induces hypoxia-like condition as reported [15], to mimic hypoxia status and investigated the role of melittin in VM of liver cancer and the underlying mechanisms.

## 2. Materials and Methods

### 2.1. Materials

Melittin (C_131_H_229_N_39_O_31_; >97% purity) was a product of Santa Cruz Biotechnology (Santa Cruz, CA, USA). It was dissolved in distilled water to make a stock solution and then stored at −20°C until use avoiding repeated freezing and thawing.

### 2.2. Cell Culture

The human liver cancer cell lines, SMMC-7721, Huh7, and Hep G2, were cultured in complete DMEM which was supplemented with 10% fetal bovine serum (FBS) and 100 units/ml penicillin, 0.1 mg/ml streptomycin (all Hyclone, Life Sciences, Logan, UT, USA) at 37°C in a humidified atmosphere of 5% CO_2_. Cells were subcultured when the cell density reached 70-80% confluence.

### 2.3. MTT Assay for Cell Proliferation

The SMMC-7721, Huh7, and HepG2 cells were seeded in 96-well culture plates with a density of 1×10^4^ cells/well. After 24 h, various concentrations of melittin in normoxia condition (0, 1, 2, 4, 6, 8 12 *μ*g/ml) or in the presence of CoCl_2_ (0, 4, 8 *μ*g/ml) were added and cultured for another 24, 48, and 72 h, respectively. Then 20 *μ*L of MTT solution (5 mg/mL) was added to each well and incubated for additional 4 h at 37°C. The medium was carefully removed and 100 *μ*l DMSO was added. After being incubated overnight, the absorbance at a wavelength of 490 nm was measured using a multiskan spectrum microplate reader. The mean cell proliferation was calculated from the absorbance units.

### 2.4. Cell Migration and Invasion Assays

Transwell cell-culture chamber (BD Biosciences, Franklin Lakes, NJ, USA) assays were used to assess the invasion and migration activity of cells as described previously [13]. Briefly, SMMC-7721, Huh7, and HepG2 cells suspended in 100 *μ*l serum-free medium were seeded into the upper chambers at a density of 1 × 10^4^/well for migration and 2 × 10^4^/well for invasion. Then 500 *μ*l complete media were added to the lower compartment. For the invasion assays, the Matrigel (BD Biosciences, Franklin Lakes, NJ, USA) was diluted with serum-free medium according to the manufacturer instructions at a ratio of 1:5 and added 100 *μ*l/well to the upper chamber sand incubated at 37°C for 1 h before seeding cells. CoCl_2_ with or without indicated concentrations (0, 2, 4 *μ*g/ml) of melittin was added to the upper compartment. After incubation at 37°C for 24 h, cells were stained with 0.1% crystal violet and images of the cells that had migrated or invaded to the lower chamber of the polycarbonate membrane were captured.

### 2.5. VM Tube Formation Assay In Vitro

The VM network was measured by the method described previously [16]. In brief, the Matrigel basement membrane matrix (BD Biosciences) stored at −20°C was thawed at 4°C overnight before use. 50 *μ*l matrix was added to each of the 96-well plates and incubated at 37°C for 2 h to solidify. The cells were trypsinized to single cell suspension and adjusted to 5×10^5^/ml. 100 *μ*l cells were seeded on the Matrigel-coated plates and CoCl_2_ with or without indicated concentrations (0, 2, 4 *μ*g/ml) of melittin was added to each well. Then cells were incubated for 24 h. Images of each well were obtained using an inverted phase contrast microscope.

### 2.6. Real Time RT-PCR

Total RNA was extracted from SMMC-7721 cells after treatment with CoCl_2_ and indicated concentrations (0, 2, 4 *μ*g/ml) of melittin with TRIzol reagent (Invitrogen, Carlsbad, CA, USA) as described previously [17]. cDNA was synthesized using a first strand cDNA synthesis kit (Takara Inc., Dalian, P. R. China). Real time PCR was performed using a SYBR Green PCR Master Mix (TOYOBO, Osaka, Japan) under the following conditions: denaturation under 95°C for 3 min and subjected to conditions of 95°C for 10 s, 60°C for 20 s, and 72°C for 25 s for a total of 40 cycles. The primers used in this study were as follows: *β*-Actin, Forward, 5′-AGC GGG AAA TCG TGC GTG -3′; Reverse, 5′-CAG GGT ACA TGG TGG TGC C-3′; E-cadherin, Forward, 5′- CCC AAT ACA TCT CCC TTC ACA G-3′; Reverse, 5′- CCA CCT CTA AGG CCA TCT TTG-3′; N-cadherin, Forward, 5′-CAA GAG GCA GAG ACT TGC GA-3′; Reverse, 5′-CAC ACT GGC AAA CCT TCA CG-3′; vimentin, Forward, 5′- CCT CAC CTG TGA AGT GGA TGC-3′; Reverse, 5′- CAA CGG CAA AGT TCT CTT CCA-3′. The relative expression level of mRNA of each sample was calculated by the 2^−∆∆Ct^ method and *β*-actin was used for normalization.

### 2.7. Western Blot Analysis

Liver cancer cells were seeded on the 6-well plates and CoCl_2_ with or without indicated concentrations (0, 2, 4 *μ*g/ml) of melittin was added to each well for the indicated time. LY294002 (obtained from Selleck, China), a PI3K inhibitor, was add into the cells 2 h before treatment of CoCl_2_ or melittin with concentration of 10 *μ*M. After 24 h of treatment of CoCl_2_ or melittin, total protein from tumor cells was isolated as described previously [18]. BCA method was used to determine the protein concentration and cell lysis was used to make all the samples in the same concentration. 30 *μ*g proteins of each sample were separated by sodium dodecyl sulfate polyacrylamide gel electrophoresis (SDS-PAGE) and then transferred to nitrocellulose (NC) membranes. After being blocked with 5% BSA in 1 × TBST buffer (20 mM Tris-HCl, pH 7.4, 150 mM NaCl and 0.1% Tween 20) for 1 h under room temperature, the NC membrane was incubated with specific primary antibodies overnight at 4°C and then a secondary antibody for 1 h under room temperature. Rabbit p-Akt and t-Akt polyclonal antibody, rabbit E-cadherin, N-cadherin and Vimentin monoclonal antibody, rabbit matrix metalloproteinase (MMP)-2 and MMP-9 polyclonal antibody (all 1:1000), mouse *β*-actin (1:5000) monoclonal antibody were all purchased from Cell Signaling Technology (Boston, MA, USA). Mouse HIF-1*α*, VEGF and VM monoclonal antibody (both 1:200) were obtained from Santa Cruz Biotechnology, Inc. (Santa Cruz, CA, USA). Rabbit (1:2000) and mouse (1:5000) secondary antibodies were purchased from Cell Signaling Technology. The immunoreactive bands were detected using an enhanced chemiluminescence kit (ECL) (Thermo, CA, USA) and imaged with G:BOX Chemi XR5 (Syngene, Frederick, MD, USA).

### 2.8. Tumor Establishment and In Vivo Treatments

SMMC-7721 cells were trypsinized and resuspended with PBS. 1×10^7^ cells were injected subcutaneously into each male BALB/c nude mice (6-8 weeks old, 20 ± 2 g, Shanghai SLAC Laboratory Animal Co. Ltd., Shanghai, China) to produce implanted tumors. Then the mice were randomly divided into three groups: Control, 50 *μ*g/kg·d melittin, and 100 *μ*g/kg·d melittin groups. Melittin (50 or 100 *μ*g/kg·d) was intravenously injected via tail vein daily. The same volume of saline was used for the control mice. Then, the volumes of tumors were measured with a slide caliper and were evaluated by the following equation: a (the larger diameter) × b (the smaller diameter)^2^/2. After 11 days of treatment, mice were anaesthetized with overdose of Pentobarbital sodium (1%) through intraperitoneal injection and were sacrificed. All procedures were performed in accordance with the Helsinki Declaration. The experiment was approved by the Ethics Committee of Second Military Medical University.

### 2.9. Immunohistochemical Staining

Immunohistochemical staining was performed according to previous description [19]. Monoclonal mouse anti-human CD34 antibody (1:100 dilution, sc-65261; Santa Cruz) and monoclonal mouse anti-human HIF-1*α* (1:100 dilution; sc-13515, Santa Cruz) were incubated with the tumor sections overnight. After being washed with PBS for 3 times, the tumor sections were incubated with rabbit anti-mouse biotinylated secondary antibody at room temperature for 15 min, followed by incubating with horseradish peroxidase (HRP)-conjuncted streptavidin for another 30 min at room temperature. Diamino-benzidine (DAB) assay was used to detect the immunoactivity.

### 2.10. CD34/PAS Double Staining

After immunohistochemical staining for CD34, the sections were washed with distilled water and incubated with periodic acid solution for 7 min. Then, Schiff solution was added to the sections for 20 minutes after the sections were washed with distilled water for 3 minutes. Schiff solution was washed away with distilled water and hematoxylin staining and gradient dehydration was applied prior to mounting with neutral gum.

### 2.11. Statistical Analyses

Statistical analysis was performed with SPSS software (Version 21.0, SPSS, Inc., Chicago, IL, USA). Data are expressed as means ± S.D. and one-way analysis of variance were used for multiple comparisons of the difference between groups followed by Student-Newman-Keuls tests. A *P* value < 0.05 indicated statistical significance.

## 3. Results

### 3.1. Melittin Inhibited the Viability, Migration, and Invasion of Liver Cancer Cells under Hypoxic Condition

**Figure 1:**
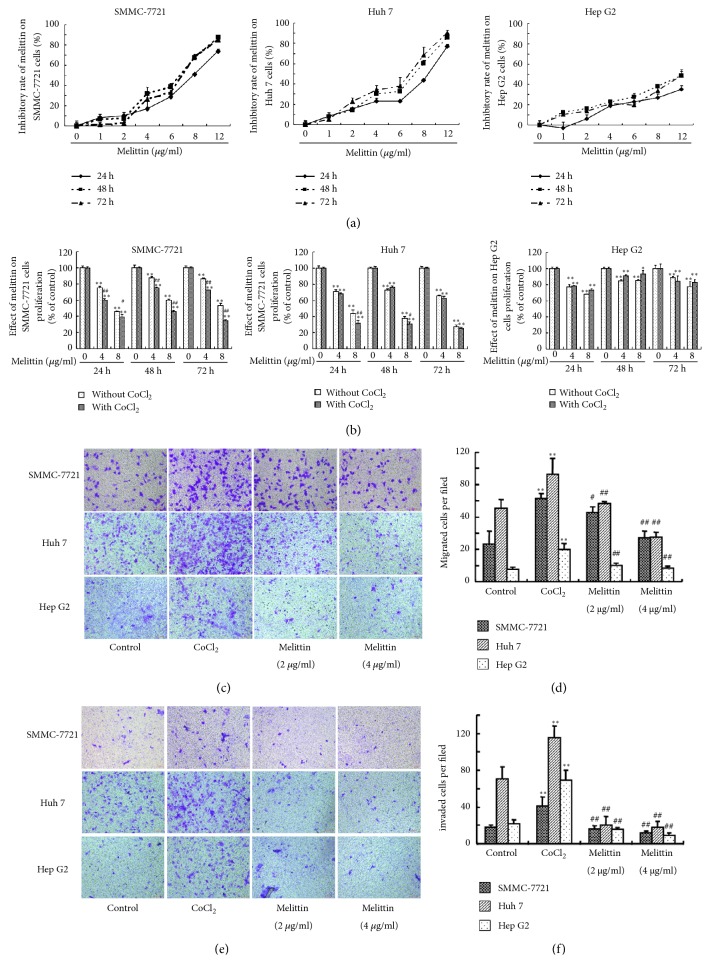
Effect of melittin on the proliferation, migration, and invasion of hepatoma cells. The inhibitory rates of melittin on the SMMC-7721, Huh7, and HepG2 hepatoma cells were determined under normal condition (a) and in the presence of CoCl_2_ (b). Then the effect of melittin on CoCl_2_-induced migration (c, d) and invasion (e, f) of SMMC-7721, Huh7, and HepG2 hepatoma cells was examined by transwell assay. SMMC-7721, Huh7, and HepG2 hepatoma cells were seeded into the upper compartment of a Transwell Boyden chamber with CoCl2 (150 *μ*M) or CoCl2 + melittin. The control group was cultured without CoCl2 and melittin. At least 6 random fields (×100) per condition were counted. Data are expressed as means ± S.D. (*N*=6) ^*∗*^*P*<0.05, ^*∗∗*^*P*<0.01 compared with control group; ^#^*P*<0.05, ^##^*P*<0.01 compared with CoCl_2_ group.

We first studied the effect of melittin on the viability of liver cancer cells. Under the normoxia condition, melittin significantly inhibited the viability of SMMC-7721, Huh7, and HepG2 cells at 24, 48, and 72 h (Figure 1(a)). The inhibitory rates of melittin on SMMC-7721, Huh7, and HepG2 hepatoma cells increased in a dose-dependent manner. In the presence of CoCl_2_, the inhibitory rates of melittin on the SMMC-7721 and Huh7 cells were further increased compared with those without CoCl_2_ (Figure 1(b)), whereas there were no significant differences in HepG2 cells with or without CoCl_2_.

It has been shown that hypoxia may facilitate the metastasis of liver cancer cells [20]. CoCl_2_ treatment significantly elevated the numbers of migrated and invaded SMMC-7721, Huh7, and HepG2 cells (Figures 1(c)–1(f)). Cotreatment with melittin significantly decreased the migrated and invaded numbers of SMMC-7721, Huh7, and HepG2 cells.

### 3.2. Melittin Inhibited the VM Formation In Vitro Induced by CoCl_*2*_

**Figure 2:**
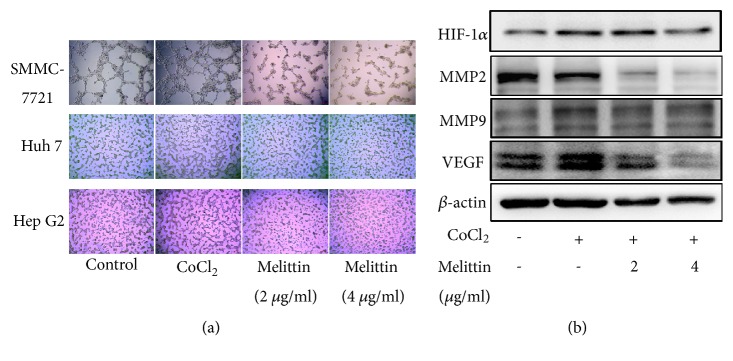
Effect of melittin on hypoxia-induced VM formation of hepatoma cells. (a) Melittin inhibited hypoxia-induced VM formation. SMMC-7721, Huh7, and HepG2 hepatoma cells were seeded into Matrigel-coated wells with CoCl_2_ (150 *μ*M) or CoCl_2_ + melittin and allowed to form tubular structures. Tubular structures were photographed 24 h later at 100 × magnification. (b) Western blot analysis of VM-related proteins.

Next, we observed the effect of melittin on the VM formation of liver cancer cells. As shown in Figure 2(a), CoCl_2_ treatment for 24 h obviously increased the formation of VM. Treatment with melittin (2 and 4 *μ*g/ml) suppressed CoCl_2_-induced VM formation.

To elucidate the mechanisms involved in the reverse effect of melittin on hypoxia-induced VM formation, we first examined the levels of HIF-1*α* after melittin treatment. As shown in Figure 2(b), CoCl_2_ treatment increased the level of HIF-1*α* and its downstream target genes VEGF and MMP-9 that are involved in VM formation and tumor invasion [21]. Melittin suppressed CoCl_2_-induced upregulation of HIF-1*α*, VEGF, and MMP-2/9 (Figure 2(b)).

### 3.3. Melittin Inhibited EMT Induced by CoCl_*2*_

**Figure 3:**
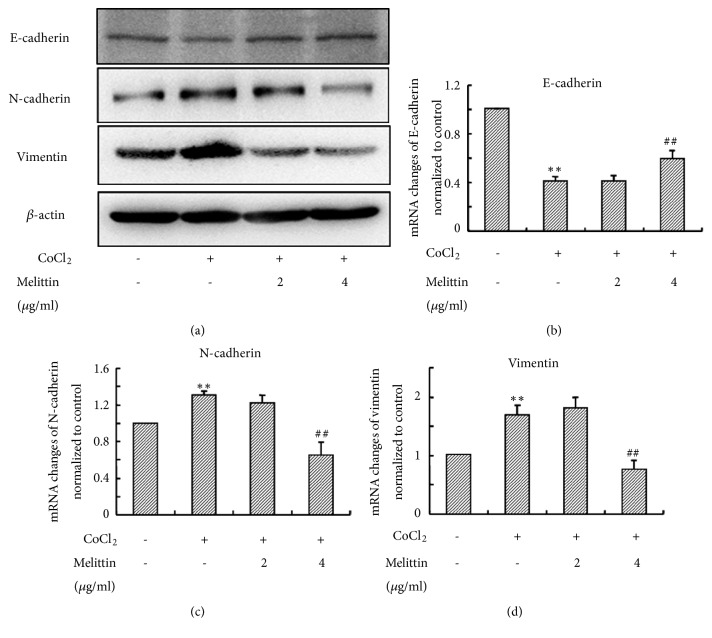
Melittin inhibits CoCl_2_-induced EMT in SMMC-7721 cells. (a) Western blot analysis of EMT markers; (b)–(d) the mRNA levels of EMT markers after CoCl_2_ treatment with or without melittin were determined by real time RT-PCR. Data are expressed as means ± S.D. (*N*=3) ^*∗*^*P*<0.05, ^*∗∗*^*P*<0.01 compared with control group; ^#^*P*<0.05, ^##^*P*<0.01 compared with CoCl_2_ group.

EMT is widely recognized to be involved in cancer invasion. Furthermore, EMT also plays a vital role in VM formation [22]. In addition, overexpression of HIF-1*α* has been shown to induce EMT of liver cancer [23]. Therefore, we further investigated the effect of melittin on hypoxia-induced EMT. CoCl_2_ treatment suppressed the expression of E-cadherin (Figure 3(a)). Consistently, the expression of N-cadherin and Vimentin were increased by CoCl_2_ (Figure 3(a)). Melittin reversed the changes in the protein levels of these EMT markers.

Subsequently, we used real time RT-PCR to quantify mRNA levels of EMT markers. In consistence with western blot results, the expression of N-cadherin and Vimentin mRNA were upregulated and the level of E-cadherin mRNA was downregulated by CoCl_2_ (Figures 3(b)–3(d)). Melittin also reversed CoCl_2_-induced mRNAs changes of EMT markers.

### 3.4. Melittin Inhibited CoCl_*2*_-Induced Activation of Akt Pathway in Liver Cancer Cells

**Figure 4:**
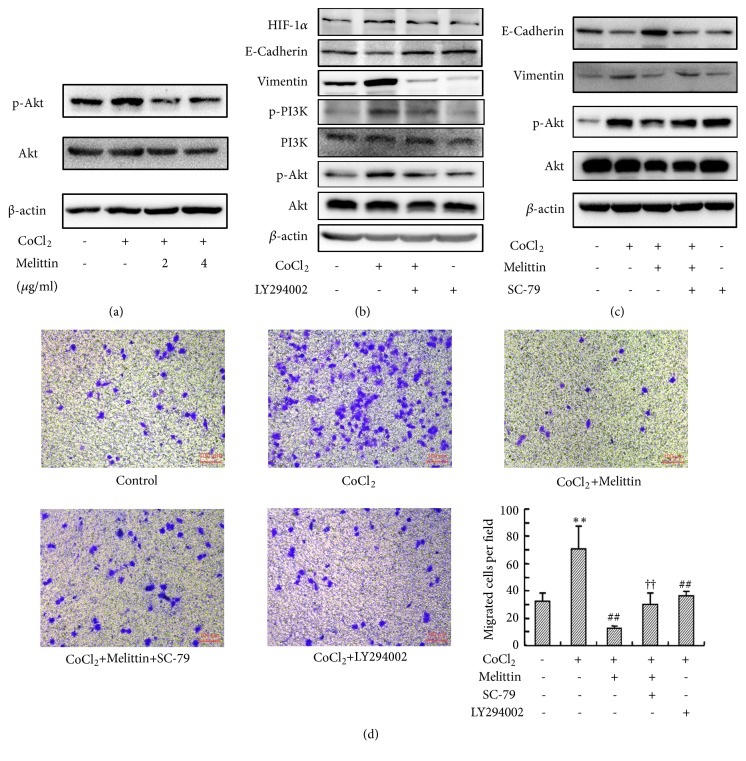
The inhibitory effect of melittin on CoCl_2_-induced EMT is mediated by Akt pathway. (a) Melittin inhibited CoCl_2_-induced phosphorylation of Akt; (b) LY294002 inhibited CoCl_2_-induced EMT; (c) SC-79 blocked the effect of melittin on CoCl_2_-induced EMT; (d) activation of Akt reversed the effect of melittin on CoCl2-induced migration of SMMC-7721. Data are expressed as means ± S. (d) (*N*=6) ^*∗∗*^*P*<0.01 compared with control group; ^##^*P*<0.01 compared with CoCl_2_ group; ^††^*P*<0.01 compared with CoCl_2_+melittin group.

Akt activation plays an important role in the VM formation and EMT of malignant tumors [24–26]. Thus, we next estimated the level of p-Akt in SMMC-7721 cells after CoCl_2_ treatment. CoCl_2_-induced accumulation of HIF-1*α* increased the level of phosphorylated Akt, which was suppressed by melittin (Figure 4(a)). To further study the role of Akt in the inhibitory effect of melittin on hypoxia-induced EMT, we then used an inhibitor of PI3K/Akt in CoCl_2_-induced EMT. LY294002 did not obviously affect the level of HIF-1*α* after CoCl_2_ treatment, whereas it reversed the effect of CoCl_2_ on the expression of EMT markers (Figure 4(b)). SC-79, an activator of Akt, abolished the inhibitory effect of melittin on hypoxia-induced EMT (Figure 4(c)), indicating melittin reverses hypoxia-induced EMT through inhibiting HIF-1*α* induced activation of Akt. Transwell assay also showed that both melittin and LY294002 significantly inhibited CoCl_2_-induced migration of SMMC-7721 cells (Figure 4(d)). Cotreatment with SC-79 significantly increased the number of transmigrated cells compared with melittin group, implying Akt activation may reverse the effect of melittin on CoCl_2_-induced migration of hepatoma cells.

### 3.5. Melittin Inhibited VM and HIF1-*α* In Vivo

**Figure 5:**
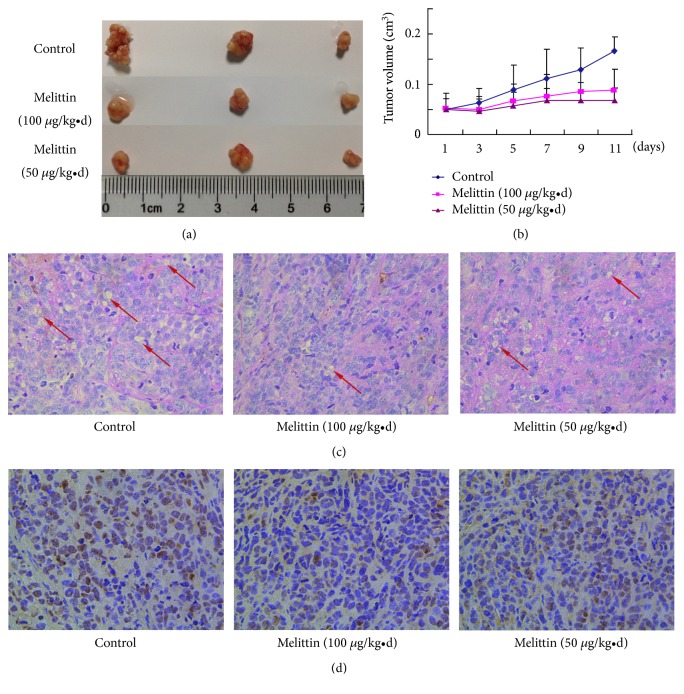
Melittin inhibits VM formation and HIF1-*α* expression in the xenografts in nude mice. (a) and (b) Melittin inhibited the tumor growth in vivo. Data are expressed as means ± S.D. (*N*=3). (c) The VM formation in the xenografts in nude mice determined by CD34 and PAS double staining. (d) Immunohistochemical staining of the HIF1-*α* expression in the xenografts in nude mice.

To verify the in vitro results of melittin on VM formation, we conducted a nude mice model of human liver cancer and treated with melittin by tail vein injection. As shown in Figures 5(a) and 5(b), melittin treatment significantly suppressed the tumor growth as reported previously. CD34 and PAS double staining showed that the VM formation in the tumor was less in the melittin treatment groups than that in control group (Figure 5(c)). Immunohistochemical staining showed that HIF-1*α* expression in the tumor was significantly suppressed by melittin (Figure 5(d)).

## 4. Discussion

VM, which is associated with high tumor grade, invasion and metastasis, and short survival, has been considered as a marker of poor clinical prognosis in liver cancer [4, 27]. Formed by aggressive tumor cells to mimic vasculogenic networks, VM plays an important role in the liver cancer malignancy as liver cancer is a typical hypervascular solid tumor [28, 29]. Although experiments indicated that melittin may exert antiangiogenic activity through VEGF pathway, whether it may inhibit liver cancer metastasis through VM has not yet been studied [30]. In the present study, we investigated the role of melittin in VM formation under hypoxic condition. Our results showed that melittin inhibited liver cancer cells proliferation, migration, and invasion under the hypoxic condition. Furthermore, melittin also inhibited the hypoxia-induced VM formation both in vitro and in vivo.

Liver cancer is a typical malignant tumor with rich blood supply. However, many treatment methods for liver cancer usually induce a hypoxia microenvironment, including TACE and radiofrequency ablation [20, 31], and thereafter promote tumor angiogenesis and metastasis, in part related to the accumulation of HIF-1*α* [32]. Overexpression of HIF-1*α* promotes the growth, migration, and invasion of liver cancer cells and induces the upregulation of VEGF, which is one of the most potent angiogenic factors presented in various human cancers [33]. In the current study, melittin inhibited CoCl_2_–induced liver cancer cells proliferation, migration, and invasion, suggesting it may inhibit the growth and metastasis induced by hypoxia.

The correlation of hypoxia and VM formation has been widely claimed in many types of malignant tumors, including liver cancer. VM formation positively correlates with the invasion and metastasis of liver cancer [4, 5, 34]. VM formation allows the tumor cells easily entering into the nearby microcirculation environment, thereby promotes the metastasis of tumor cells to other organs, and thus plays a crucial role in the tumor metastasis [35]. Our data showed that melittin suppressed the VM formation of liver cancer cells after CoCl_2_ treatment. Hypoxia is also able to induce EMT, which allows cancer cells transdifferentiating into mesenchymal cells, resulting in the acquisition of invasive and metastatic properties [36]. It has been demonstrated that EMT plays a crucial role in VM formation [37]. Our results also showed that melittin inhibited CoCl_2_-induced EMT of liver cancer cells. Interestingly, it seems that low dosage of melittin does not affect translational level of vimentin, indicated by the result such that 2 *μ*g/ml of melittin obviously inhibited the level of vimentin protein, but only 4 *μ*g/ml of melittin significantly suppressed the level of vimentin mRNA, which was shown in Figure 3 and need to be further explored. In summary, the above results suggest the inhibition of melittin on the VM formation of liver cancer cells may be related to the suppression of EMT.

The proliferation and motility, including migration, invasion, and adhesion are two key elements for the formation of VM channels by tumor cells. In addition, the activation of Akt is required for cells proliferation, migration, and tube-like structure formation [38, 39]. VEGF, which plays an important role in the formation of VM, is upregulated by HIF-1*α* and thereby activates the PI3K/Akt pathway through its receptors. Interestingly, inhibiting the PI3K/Akt pathway also suppresses the expression of HIF-1*α* and VEGF [40], suggesting Akt pathway plays a central role in the induction of VEGF and formation of VM. In current study, our results showed that melittin decreased CoCl_2_-induced HIF-1*α* accumulation, VEGF overexpression, and Akt phosphorylation in SMMC-7721 cells. Furthermore, MMPs serve as important downstream effectors of VEGF and Akt and participate in cell motility and VM formation [41, 42]. In the current study, melittin also suppressed the MMP2/9 expression. In addition, the effect of melittin on CoCl_2_-induced EMT and migration of liver cancer cells was reversed, at least partly, by the activator of Akt, suggesting that melittin inhibits hypoxia-induced EMT and VM formation through HIF-1*α*/Akt pathway. Consistent with the findings in vitro, treatment with melittin greatly reduced the protein expression of HIF-1*α* and decreased the formation of VM in the xenografts in nude mice. Interestingly, we also noted that the effect of melittin at 50 ug was a little better than 100 *μ*g. However, there are no significant differences between the two groups at each time point. This may be due to the fact that the effect on melittin has hit the plateau at 100*μ*g in suppressing the growth of tumor in vivo. However, in the present, we mainly focused on the effect of melittin on the hypoxia-induced VM formation in liver cancer. We may deduce the conclusion from our results that melittin is able to inhibit the VM formation and HIF-1A in vivo.

There are also some limits in the present study; in the future studies, we will further investigate the role of HIF-1*α* and Akt in the effects of melittin on liver cancer cells using RNA interference. On the other hand, recent study showed that analog of melittin such as MEL-pep may show better effect since it may perform a promising role on chemotherapy resistant liver cancer [43]. However, challenges of applicability of melittin to humans are relate to several issues including its degradation and hemolytic activity. Therefore, further studies about better analogs of melittin or utilization of nanoparticle based delivery of melittin is extremely essential to exert its applicable anticancer effect. [44]

## 5. Conclusions

In summary, this study elucidates a new mechanism of the antiliver cancer effect of melittin through inhibiting hypoxia-induced VM formation. Suppression of EMT and inhibition of HIF-1*α*/Akt pathway are implicated to be involved in the inhibitory effect of melittin on hypoxia-induced VM formation. However, how melittin affects the synthesis and stability of HIF-1*α* should be further investigated in the future.

## Data Availability

The data used to support the findings of this study are available from the corresponding author upon request.
